# Advantages of Using Extremophilic Bacteria for the Biosynthesis of Metallic Nanoparticles and Its Potential for Rare Earth Element Recovery

**DOI:** 10.3389/fmicb.2022.855077

**Published:** 2022-03-21

**Authors:** Joaquín Atalah, Giannina Espina, Lotsé Blamey, Sebastián A. Muñoz-Ibacache, Jenny M. Blamey

**Affiliations:** ^1^Fundación Biociencia, Santiago, Chile; ^2^Facultad de Química y Biología, Universidad de Santiago de Chile, Santiago, Chile

**Keywords:** *Geobacillus*, thermophiles, bionanoparticles, enzymes, lanthanide, lanmodulin

## Abstract

The exceptional potential for application that metallic nanoparticles (MeNPs) have shown, has steadily increased their demand in many different scientific and technological areas, including the biomedical and pharmaceutical industry, bioremediation, chemical synthesis, among others. To face the current challenge for transitioning toward more sustainable and ecological production methods, bacterial biosynthesis of MeNPs, especially from extremophilic microorganisms, emerges as a suitable alternative with intrinsic added benefits like improved stability and biocompatibility. Currently, biogenic nanoparticles of different relevant metals have been successfully achieved using different bacterial strains. However, information about biogenic nanoparticles from rare earth elements (REEs) is very scarce, in spite of their great importance and potential. This mini review discusses the current understanding of metallic nanoparticle biosynthesis by extremophilic bacteria, highlighting the relevance of searching for bacterial species that are able to biosynthesize RRE nanoparticles.

## Introduction

Metallic nanoparticles (MeNPs) possess unique physical properties that make them highly attractive in material science and biology. Their nanometric scale is comparable in size to subcellular structures, and the wide variety of core materials available, coupled with tunable surface properties, make nanoparticles an excellent platform for a broad range of notable applications (De et al., 2008). Due to the increasing interest, back in 2018, market reports estimated that the worldwide production of MeNPs had the expectation of reaching 50 billion US dollars by 2026 (Ovais et al., 2018). However, amid the COVID-19 crisis, healthcare applications of nanoparticles are storming into the spotlight, led by the focus on diagnostics and treatment of SARS-CoV-2 (Brouwer et al., 2021; Park et al., 2021; Pilaquinga et al., 2021; Wang et al., 2022) as well as other viruses (Draz et al., 2020; Dung et al., 2020; Perotti and Perez, 2020) and bacterial infections (Barani et al., 2021). Therefore, the global market for nanotechnology is currently projected to keep growing (Rasmi et al., 2021; Tortella et al., 2021), and will most probably surpass the pre-pandemic estimation.

Biomedical applications take advantage of the small size of nanoparticles, which allows them to easily surpass the blood–brain barrier and skin epithelium, making them suitable as drug-carriers. Additionally, the high surface area-to-volume ratio of MeNPs in solution and their unique chemical and physical properties such as uniformity and conductance, improves the distribution of the drugs being delivered, enhancing its therapeutic impact (Khan et al., 2018). Also, the functionalization of MeNPs with various macromolecules has been proven to expand and further improve many biomedical properties. Their hybridization with biopolymers such as chitosan, hyaluronic acid, cellulose, or starch has shown betterment in release kinetics, biocompatibility, and pharmacokinetics of drug-carrier MeNPs (Hussain et al., 2019).

Several MeNPs have also shown to exert antibacterial, antifungal, and antiviral effects, and they are currently used in preventing biofilm formation and antibiotic resistance (Fernando et al., 2018; Gahlawat and Choudhury, 2019; Thambirajoo et al., 2021). A study working with clinical *Acinetobacter baumannii* isolates, that showed high level of resistance to antibiotics, proved that treating the bacteria with silver nanoparticles (AgNPs) markedly interrupted bacterial growth, inhibited biofilm formation, and downregulated transcription levels of important virulence genes (Hetta et al., 2021). In addition, MeNPs have also gained attention for their antioxidant properties, with potential application in pharmaceutical and cosmetic products (Patel et al., 2021). Furthermore, nanoparticles applications also harness extraordinary potential to contribute to environmental protection amid the climate change crisis, as they can be used for bioremediation, and also as additives to enhance the efficiency of biofuels and pesticides, reducing their use (Johnson et al., 2013; Rai et al., 2018; Shang et al., 2019; Chauhan et al., 2022).

A variety of chemical and physical methods has been used for the synthesis of MeNPs. However, these conventional methods are fraught with many problems including its cost, high-energy consumption, the use of toxic solvents, and the generation of hazardous by-products, which affects the biocompatibility of MeNPs, limiting their applications (Thakkar et al., 2010). In addition, the scalability of the traditional methods is also challenging because of the low stability and monodispersity of the obtained products (Ali et al., 2019). Accordingly, there is an increased pressure and demand to develop clean, nontoxic, eco-friendly, and economical methods of MeNPs synthesis (Hosseini and Sarvi, 2015).

A promising approach to achieve a more efficient and sustainable MeNPs production method is to take advantage of the array of microbiological resources in nature, as microorganisms play an important role in adsorbing and accumulating metals from their environment (Velusamy et al., 2016). In order to survive at high metal concentrations, they reduce its toxicity by changing the redox state of the metallic ions present in their surroundings, which in some cases leads to the formation of well-defined metallic nanoscale particles.

In this minireview, we aimed to present recent information related to bacterial biosynthesis of MeNPs and the advantages of using extremophilic bacteria, emphasizing the relevance of rare earth bionanoparticles.

## Extremophilic Bacteria in the Biosynthesis of Nanoparticles

Producing MeNPs using biotechnological processes is usually less costly, highly efficient, and more ecological than chemical methods. Several bacteria possess remarkable ability to reduce metal ions, and are one of the best candidates for production of biogenic nanoparticles. Bacterial NPs biosynthesis has the advantage of producing more stable MeNPs that show a lowered tendency toward agglomeration. This improved stability is believed to be caused by the capping of the NPs surface by native microbial proteins and other biomolecules (Ovais et al., 2018). These capping proteins bind to MeNPs through their amine groups and cysteine residues, neutralizing the net surface charge of the particles, preventing agglomeration which would cause the loss of their specific properties. In the case of chemically produced NPs, oftentimes surfactants are used to increase stability, but these compounds are usually toxic, hindering their biomedical applications. Protein-enabled stability of biosynthesized NPs is non-toxic, environmentally friendly, and overall safer (Beeler and Singh, 2016).

Furthermore, MeNPs biosynthesis by bacteria offers greater control over the conditions of the production process, which is relevant given that pH, the medium used, presence of light, concentration of metal ions, and temperature could affect the shape, size, and manufacture efficiency of MeNPs. Because of this, extremophilic microorganisms that are naturally adapted to live under extreme environmental conditions (e.g., high metal concentration, high and low temperature, acidic or alkaline pH, high pressure, salinity, and radiation) are particularly relevant to MeNPs biosynthesis. Many extremophiles have specific mechanisms to quell stresses associated with metal toxicity, and some of them grow optimally at very high metal ion concentrations, such as those found in hydrothermal vents or volcanic areas, dumping sites of metallurgical waste or mining waste from processed ores, where mesophilic microorganisms simply cannot survive. Some of them are even able to live under multiple stress conditions, reason why they are recognized as polyextremophiles, such as thermophilic and acidophilic microorganisms that also possess very high metal resistance.

As extremophiles thrive in a wide variety of harsh conditions, their use allows for greater control of the MeNPs production parameters. They also produce robust and stable capping proteins that resist harsh conditions (Beeler and Singh, 2016), resulting in MeNPs of higher chemical stability (Correa-Llantén et al., 2013). *Deinococcus radiodurans* is a prime example of the usefulness of extremophiles in MeNPs synthesis. This polyextremophile is an extremely radioresistant, thermotolerant, psychrotolerant, and acid tolerant bacterium that has been shown to produce protein capped spherical silver NPs. Interestingly, the size of the AgNPs produced decreased as the concentration of silver chloride supplemented to the reaction media increased (Kulkarni et al., 2015). This kind of control over the size of the NPs produced is a unique feature of biosynthesis. Besides the concentration of the salt precursors, other factors such as pH, temperature, and reaction time are crucial variables determining NPs size (Ovais et al., 2018).

Other bacteria show similar features that allow for NPs size control. For example, the thermophilic bacterium *Geobacillus wiegelii* strain GWE1, which was isolated from a drying oven, was shown to produce non-metallic nanoparticles. When exposed to Na_2_TeO_3_ salt the microorganism was able to produce elemental tellurium NPs and when exposed to Na_2_SeO_3_, was able to reduce Se^4+^ to Se^0^ to form selenium NPs, both intra and extracellularly. The size and shape of Se-NPs produced by this microorganism has been shown to be modulated by pH and temperature (Correa-Llantén et al., 2014), and the size of the NPs was further modified when 5% polyvinylpyrrolidone was added to the culture medium, resulting in 52% smaller NPs (Muñoz-Ibacache et al., 2015).

Another thermophile from the same genus, *Geobacillus* sp. ID17, was shown to have the ability to intracellularly biosynthesize gold NPs. This was observed in whole cells as well as in cell extracts, which led to the finding that the biosynthesis is enzymatically mediated and requires NADH as cofactor (Correa-Llantén et al., 2013). In this context, the reported evidence that proteins and specifically some metallo-enzymes from extremophiles might play a key role in the nucleation and crystal growth of bacteriogenic MeNPs, is particularly relevant for its technological implications (Correa-Llantén et al., 2014).

## Bacterial Mechanisms of NPs Biosynthesis

Bacteria usually have specific mechanisms, which rely on their survival strategies to deal with hazardous inorganic materials in the environment, most probable as a way of stress response (Ghosh et al., 2021). Metal ions are accumulated on the surface, or inside bacterial cells, where they are reduced into MeNPs by biological reducing agents. The latter are highly variable but generally consist of reductase enzymes, exopolysaccharide, or electron shuttle quinones (Gahlawat and Choudhury, 2019; Patel et al., 2021).

The location of MeNPs biosynthesis also varies greatly. Bacteria that favor intracellular MeNPs production, can form MeNPs in the periplasmic space, in the cell wall, or in the cytoplasm. Metal ions can enter bacterial cells through ion channels, active transport, endocytosis, or through the lipid membrane (Tsekhmistrenko et al., 2020). Then, NPs formation involves metal excretion/accumulation across membranes, enzymatic action, efflux pump systems, binding at peptides, and precipitation (Ghosh et al., 2021).

Bacteria that produce extracellular MeNPs can form them at the outer surface of the cell wall or throughout the culture media, often utilizing cell-wall-associated biomolecules (Beeler and Singh, 2016). Extracellular synthesis involves enzyme secretion and particle capping (Singh and Singh, 2019). Cell walls can have a role either in intracellular or extracellular biosynthesis, and it has been found that metal ions are attracted to their negatively charged functional groups, where they nucleate before the reduction that results in MeNPs biosynthesis (Beeler and Singh, 2016).

Different enzymes can be involved in the generation of NPs, most of them belonging to oxidoreductases, a wide class of enzymes capable of catalyzing redox reactions, transferring electrons from an electron donor to an oxidant, which in the case of MeNPs production corresponds to the inorganic ions. Notably, MeNPs biogeneration can take place in the presence of specific enzymes, without requiring the presence of whole microorganisms, indicating that the full bacterial metabolic machinery is not needed (Correa-Llantén et al., 2014). Evidence has shown that NADH-dependent nitrate reductase is one of the main reducing agents in the transformation of ions into nanoparticles, particularly in the case of silver (Tsekhmistrenko et al., 2020). Bacterial synthesis of MeNPs from other metals seem to rely on similar enzymes. It has been observed that in Au-NPs production, the reduction of gold by bacteria is carried by NADH-dependent reductases. When analyzing biosynthesis of Au -NPs, the NADH-dependent Au^3+^ reductase activity found in crude extracts of *Geobacillus* sp. ID17 dropped in approximately 98% in the presence of SDS or proteinase K, reflecting the enzymatic nature of the reaction (Correa-Llantén et al., 2013).

The previously mentioned *G. wiegelii* strain GWE1 also uses NADH-dependent enzymes. MALDI-TOF analysis allowed the identification of one of the enzymes implicated in the biosynthesis process as a 1-pyrroline-5-carboxylate dehydrogenase (Correa-Llantén et al., 2014). While the halophilic bacterium *Halomonas salina*, is known to secrete the cofactor NADH and NADH-dependent enzymes, specially nitrate reductase, which is speculated to act as a scaffold or nucleating agent that might be responsible for the bioreduction of Au^+3^ to Au^0^, and subsequent formation of thermodynamically stable gold nanoparticles, which is pH and temperature dependent (Shah et al., 2012).

Not only NADH-dependent reductases are involved in NPs biosynthesis. An interesting example is the ABC-transport protein capable of reducing gold ions in Au-NPs synthesis by the thermophilic bacterium *Thermus scotoductus* SA-01 (Erasmus et al., 2014). The authors speculate that this protein reduces Au^3+^ through an electron shuttle mechanism through a cysteine disulfide bridge with no cofactors involved. The protein also showed to initiate nucleation of the ions for MeNPs synthesis.

Beside enzymes, several biocompounds can be used by bacteria for the production of MeNPs, including naphthoquinones, hydroquinones, and anthraquinones (Ovais et al., 2018). The extracellular polymeric substances (EPS) secreted by many bacteria has also been shown to be involved in the production of MeNPs, by using its constituent saccharides as reducing agents. Au^3+^ reduction has been proven to be mediated by the hemiacetal groups (aldehyde equivalents) present in EPS (Kang et al., 2017). Other mechanisms for the synthesis of MeNPs include changes in solubility of the metal ions, biosorption, complexation, and extracellular precipitation (Ovais et al., 2018).

## Rare Earth Bionanoparticles, Recent Advances, and Current Challenges

Rare earth metals are a group of 17 chemical elements of the periodic table: lanthanum (La), cerium (Ce), praseodymium (Pr), neodymium (Nd), promethium (Pm), samarium (Sm), europium (Eu), gadolinium (Gd), terbium (Tb), dysprosium (Dy), holmium (Ho), erbium (Er), thulium (Tm), ytterbium (Yb), lutetium (Lu), scandium (Sc), and yttrium (Y). Their fundamental properties make them crucial for the development of the modern technological industry and they are currently used in rechargeable batteries, cell phones, TVs, computers, electric motors, wind turbines, among others (Balaram, 2019). Consequently, they are among the most critical elements and the European Commission of Enterprise and Industry included them in a list of materials with high supply risk and high economic importance (Patel et al., 2021).

These rare earth elements (REE) are sourced primarily from mineral ores, which are limited and unevenly distributed worldwide, and nowadays most of the production is located in only a few countries such as China, United States, Australia, and India (Balaram, 2019). Therefore, in order to ensure their supply, it is becoming urgent to find more sources, such as new deposits, or old mines (Wei et al., 2019) as well as efficient methods for recycling electronic and industrial wastes (Deshmane et al., 2019).

Recently, it has been shown that is possible to recover REE from sulfide mines left over, which is comprised of non-valuable sulfide minerals that generate acid mine drainage (AMD) when exposed to oxygen and water. This AMD forms mainly in coal and metal ore mining areas, whether active or abandoned, and generates a long-term pollution threat. A combination of electrochemical, biological, and physical methods has shown promising REE recovering rates from AMD, surpassing conventional solvent-based methods, which have minimal yields when directly applied to AMD (Patel et al., 2021). Recent works show that certain bacteria adsorb lanthanides on their surface, where they can later be detached from washing the cells with solutions of varying pH. A Gram-negative bacterium of the genus *Roseobacter* was shown to be able to adsorb every lanthanide, and the authors were able to separate them using decreasing pH washes (Bonificio and Clarke, 2016). This kind of approach holds promise for the development of biological, efficient, and environmentally friendly ways of recovering REE from AMD.

Rare earth elements can also be recovered biologically taking advantage of REE-NPs producing organisms. To date, REE-NPs have been biosynthesized almost exclusively using plant biomass. Cerium NPs, for example, have been produced using several plant extracts (Singh et al., 2020). *Petroselinum crispum* aqueous extract has been shown to synthesize CeO_2_ nano- and microparticles (Korotkova et al., 2019). Cerium oxide nanoparticles can also be obtained from *Acalypha indica* leaf extract (Kannan and Sundrarajan, 2014). These biosynthesized CeO_2_ NPs have showed antibacterial activity against Gram-positive and Gram-negative bacteria.

Other REE-NPs have been obtained from different organisms. Clove bud extract (*Syzygium aromaticum*) was used to develop an eco-friendly route for the synthesis of dysprosium oxide NPs. These NPs appear to be non-toxic, and showed no inhibition of bacterial growth when assayed against *Staphylococcus aureus* (Gram-positive) and *Escherichia coli* (Gram-negative). They were evaluated as a non-toxic system for the detection of the organic pollutant Picric Acid (Jain et al., 2021). The synthesis of lanthanide-NPs has also been carried out using *Mutingia calabura* leaf extract (Kumar et al., 2020). This La-NPs showed antioxidant activity, as well as moderate antibiotic activity against various bacterial species. They were also effective in Coomassie brilliant blue dye degradation beneath the sunlight. Terbium oxide NPs biosynthesis has been carried out incubating Tb_4_O_7_ in the presence of *Fusarium oxysporum*, a fungal species. The NPs thus obtained showed high biocompatibility and inhibited the growth of bone cancer cells, with no toxicity against normal osteoblasts even in relatively high concentrations (Iram et al., 2016).

Even though bacterial NPs biosynthesis shows enormous advantages, as discussed in previous sections, reports for the obtention of bacterial REE-NPs are almost non-existent at present. A relatively recent study achieved the biosynthesis of europium selenide (EuSe) NPs using a recombinant *E. coli* expressing phytochelatin synthase and metallothionein, both known as heavy-metal binding proteins (Kim et al., 2016). The authors speculate that the cationic precursors of the EuSe-NPs attach to the metal binding protein surfaces, aggregating into NPs through electrostatic attraction. They additionally performed *in vitro* studies using HeLa, SKOV-3, and 293T cells, showing that the EuSe-NPs obtained are cytotoxic against cancer cell lines.

The latter study shows that bacteria expressing REE binding proteins should be studied as candidates for REE-NPs production, although the use of recombinant *E. coli* or other mesophilic strains would not be suitable for real life application in the hostile conditions of AMD, where REE recovery is crucial. In this scenario, extremophiles are the ideal candidates for biomining REE from AMD. In this line, a recent publication investigated the bioaccumulation of europium by the thermophilic bacterium *Thermus scotoductus* SA-01, showing through TEM-EDX analysis, that the bacterium accumulates Eu both intracellularly and extracellularly (Maleke et al., 2019). FT-IR analysis showed that phosphate, carboxyl, and carbonyl groups from amides were involved in the biosorption of Eu, but no NPs production was reported.

A relevant protein in the bacterial bioaccumulation of REE seems to be Lanmodulin (LanM). This protein forms highly stable and water-soluble complexes with REE over a wide pH range. Recent studies show that it is the most REE-selective protein characterized to date, while retaining REE binding in the presence of industrially relevant concentrations of competing ions (Deblonde et al., 2020). It has been shown to be more selective toward REE ions than synthetic small-molecule chelators, and appeared to be stable under acidic conditions and high temperatures. It was first described in the Gram-negative *Methylorubrum extorquens*, a bacterium that has been shown to use La^3+^ as a cofactor for its methanol dehydrogenase (Nakagawa et al., 2012). LanM could be an interesting protein to consider when searching for potential REE-NPs producing bacteria.

## Conclusion and Future Perspectives

The incredibly wide array of different applications that MeNPs have in many different areas, has prompted interest in their large-scale production, which possesses the challenge of developing economic and ecologically friendly ways of obtaining them. Biosynthesis using extremophilic bacteria is an alternative that fulfills the requisites for sustainable production. It also offers several benefits over other methods of biosynthesis, including better stability toward aggregation and oxidation of the formed MeNPs, and the possibility of exploring many different reaction conditions owing to the diversity of environments bacteria survive and function in. This is why extremophilic bacteria, which have adapted to thrive in the most demanding conditions for life are ideal candidates to form MeNPs under a wide diversity of pH and temperature values, high ion concentration, or even pressures. This allows extremophiles to perform MeNPs biosynthesis in the harshly acidic, high temperature, and high oxidative stress conditions of mining sites, unlocking the opportunity to treat mining wastes *in situ*, and recover high value materials from what is otherwise debris. Bacterial biosynthesis, however, has been seldom reported for REE-NPs, despite the great interest of these elements, and the opportunities that REE-NPs forming bacteria could bring to the biomining of these scarce and valuable metals. The identification of bacterial proteins involved in the transport and biological interaction with REE, such as lanmodulin, may lead the path to discovering REE-NPs biosynthesizing thermophilic bacteria.

## Author Contributions

JA and GE: writing original draft, writing—review and editing, and funding acquisition. LB and SM-I: writing—review and editing. JB: resources, writing—review and editing, and funding acquisition. All authors contributed to the article and approved the submitted version.

## Funding

This work was supported by the projects: United States Army DEVCOM-Americas and DEVCOM ARL award W911NF-21-2-0073, AFOSR FA9550-13-1-0089, Grant G04-09 from Instituto Antártico Chileno (INACH), and Fundación Científica y Cultural Biociencia.

## Conflict of Interest

The authors declare that the research was conducted in the absence of any commercial or financial relationships that could be construed as a potential conflict of interest.

## Publisher’s Note

All claims expressed in this article are solely those of the authors and do not necessarily represent those of their affiliated organizations, or those of the publisher, the editors and the reviewers. Any product that may be evaluated in this article, or claim that may be made by its manufacturer, is not guaranteed or endorsed by the publisher.
